# Creep Monitoring of Submersible Observation Windows Using Mueller Matrix Imaging

**DOI:** 10.3390/ma16134733

**Published:** 2023-06-30

**Authors:** Haibo Tu, Xingying Bu, Ran Liao, Hailong Zhang, Guoliang Ma, Hening Li, Jiachen Wan, Hui Ma

**Affiliations:** 1 Department of Physics, Yangtze University, Jingzhou 434100, China; 2 Shenzhen Key Laboratory of Marine IntelliSensing and Computation, Guangdong Research Center of Polarization Imaging and Measurement Engineering Technology, Institute for Ocean Engineering, Shenzhen International Graduate School, Tsinghua University, Shenzhen 518055, China; 3 Institute of Deep-Sea Science and Engineering, Chinese Academy of Sciences, Sanya 572000, China

**Keywords:** creep, monitoring, Mueller matrix imaging, submersible, observation window

## Abstract

Safety of the observation window is one of the core concerns for manned submersibles. When subjected to underwater static pressure, extrusion and creep deformation always occur in the observation window, which can pose a threat to both safety and optical performance. To assess the deformation, real-time and non-contact monitoring methods are necessary. In this study, a conceptual setup based on the waveplate rotation and dual-DoFP (division of focal-plane polarimeter) polarization camera is built for the observation window’s creep monitoring by measuring the Mueller matrix images of the samples under different pressures and durations. Then, a series of characteristic parameters, such as *t*_1_, *R*, *r*, *R*′, are extracted from the Muller matrix images by Mueller matrix transformation (MMT), Mueller matrix polar decomposition (MMPD), correlation analysis and phase unwrapping method. The results demonstrate that these parameters can effectively describe the observation window’s creep at different pressure levels which are simulated by finite element analysis. Additionally, more characterization parameters, such as *ψ*, *A* and *D*, are given from the Mueller matrix images and discussed to illustrate the method’s potential for further applications and investigations. Ultimately, future devices based on this method could serve as a valuable tool for real-time and non-contact creep monitoring of the submersible observation windows.

## 1. Introduction

The observation windows play a vital role in enabling manned submersibles to observe the external environment, and the windows’ structural integrity and reliability are crucial factors in determining the submersible’s safety. Typically, major observation windows are made of polymethyl methacrylate (PMMA) material [1] which exhibits nonlinear viscoelastic properties [2] when it works at sea temperature [3] (−2 °C to 30 °C [4]). When the material is subjected to a certain constant external force, this macroscopically manifests as the continuous deformation (including the internal structure and surface) over time, which is terminologically called creep [5]. Microscopically when the material is pressurized, its internal polycrystalline structure along different angles will rotate in the direction of the applied force and present an orientation distribution [6]. This means that the molecules are re-aligned and the spacing between crystals increases [7], which is always described as the stress birefringence in optics. Therefore, for the observation windows made of this material, the creep will definitely occur when the submersibles dive in the deep sea, which would potentially affect the safety performance of the observation window [8] and alter its optical properties [9]. Therefore, it is necessary to measure and monitor the observation window’s creep of the submersible working in the deep sea.

Over the last decades, research has been conducted to monitor the observation windows’ creep, such as the works of Liu et al. [8] and Wang et al. [10], who used the strain gauges or the displacement sensors to trace the surface deformation of the windows. These methods are limited to the surface and subjected to the possible damage of the window, and only the creep strain on the window’s surface can be measured. Most researchers prefer to use the numerical modeling [11,12,13] to simulate and analyze the observation windows’ creep, including the internal stress and deformation. However, they cannot predict the actual operation of submersibles, nor can they respond to unexpected situations underwater. Thus, it is imperative to develop a real-time, non-contact method for dynamically monitoring of the observation windows’ creep.

Polarization is a critical property of light, and changes in its states can reflect sample’s microscopic physical structures at subwavelength levels [14]. The Stokes vector is commonly used to describe the state of polarized light, denoted as $S={\left[I,Q,U,V\right]}^{T}$. When light passes through a sample, the initial Stokes vector, *S_in_*, and the output Stokes vector, *S_out_*, can be connected using a 4 × 4 Mueller matrix *M* that contains the sample’s polarization properties. The relationship between them is expressed as Equation (1) [15],

(1) $$S_{in}=\left[\begin{array}{c} I_{in}\\ Q_{in}\\ \begin{array}{c} U_{in}\\ V_{in} \end{array} \end{array}\right],S_{out}=\left[\begin{array}{c} I_{out}\\ Q_{out}\\ \begin{array}{c} U_{out}\\ V_{out} \end{array} \end{array}\right],M=\left[\begin{array}{cccc} m_{11} & m_{12} & m_{13} & m_{14}\\ m_{21} & m_{22} & m_{23} & m_{24}\\ m_{31} & m_{32} & m_{33} & m_{34}\\ m_{41} & m_{42} & m_{43} & m_{44} \end{array}\right],S_{out}=M\times S_{in}.$$

In recent studies, researchers have utilized the polarized light imaging methods to characterize the optical birefringence and further analyze the stress and strain inside transparent samples like the observation windows of the submersibles. Ajovalasit et al. used the phase-shifting method to measure the residual stress of a glass sheet with a thickness of 8 mm based on photoelasticity [16]. Almeida et al. proposed a phase-shifting technique with phase-step and analyzed the stress of the annular disk under diametric compression [17]. Zhang et al. measured the stress birefringence distribution curve of flat glass by rotating a polarization detector based on polarization interferometric method [18]. Gao et al. used a polarized camera to achieve a four-step phase shift and obtained headlight lens’ stress retardation and direction images derived from Stokes matrix and Mueller matrix [19]. However, these methods rely on stress birefringence that requires a thin sample to assume a constant thickness, which is not ideal for the submersible observation windows that are typically thick enough to withstand underwater pressure. Li et al. measured the Mueller matrix of a conical observation window and characterized the stress changes during loading and unloading using specific polarization parameters [20], but she did not consider the window’s creep when a constant pressure was exerted on it. Moreover, the creep monitoring requires more attention to the balance between parameters’ sensitivity and robustness than the experiments in the pressurization since the material changes during the creep are temporally continuous but imperceptible. Despite these efforts, monitoring the observation window’s creep using polarized light techniques remains largely unexplored.

In this study, we propose a Mueller matrix imaging method to monitor the creep of scaled-down samples of observation windows, which enables a real-time, non-contact monitoring of the samples and could help enhance the submersible’s safety in the future. A pressurization device based on a jack is constructed to exert the pressure on the sample and last for 360 min. An experimental setup is built to continuously measure the sample’s Muller matrix images during the creep under different pressures. After that, some polarization parameters and the correlation coefficient derived from Mueller matrix images are shown to characterize the sample’s creep under different pressures. The finite element analysis is used to verify the reliability of the above characteristic parameters. Furthermore, additional polarization parameters are given and discussed to further understand the creep and physical properties of the sample. Our work suggests a real-time and non-contact polarized imaging technique to monitor the observation window’s creep, which has the potential to contribute to the characterization of creep behavior of some transparent materials like PMMA. 

## 2. Materials and Methods

### 2.1. Material

**Figure 1:**
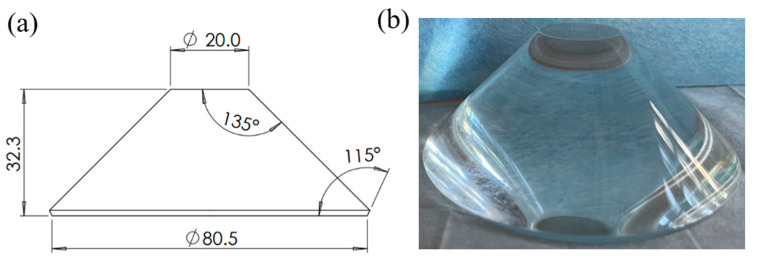
(**a**) Dimensional drawing and (**b**) physical drawing of the sample. (unit: mm).

A scaled-down sample manufactured by Tiemao Glass (Nantong, China) is made to replicate the material and shape of a real observation window in Stachiw et al. studies [1]. The engineering drawing and physical object of the conical sample are shown in Figure 1a,b, respectively. This sample has a former surface with an 80.5 mm diameter, a latter surface with a 20 mm diameter, a thickness of 32.3 mm, and a complementary taper angle of 135°. It is made of PMMA material, which boasts good light transmission, mechanical strength, and impact resistance. The creep curve of the material may exhibit three stages [21]: the first stage (primary creep) refers to the deformation stage after instantaneous strain, during which the creep rate continually diminishes due to the increase of creep resistance; this is followed by the second stage (steady-state creep), during which the creep rate decreases to a minimum and tends to be constant due to the balance between strain hardening and thermal softening; the third stage (tertiary creep) is the accelerated creep stage, during which the creep strain rate rapidly increases and eventually results in failure. In addition, the shape of the creep curve is related to stress, and higher stress levels will accelerate creep deformation [5]. Table 1 shows the physical properties of this material, which are given by Tiemao Glass and refer to Li et al.’s studies [20]. Generally, based on Table 1, if the geometry of the sample and its environmental temperature and pressure are given, one can theoretically predict the creep curve of this sample.

### 2.2. Experimental Setup

**Figure 2:**
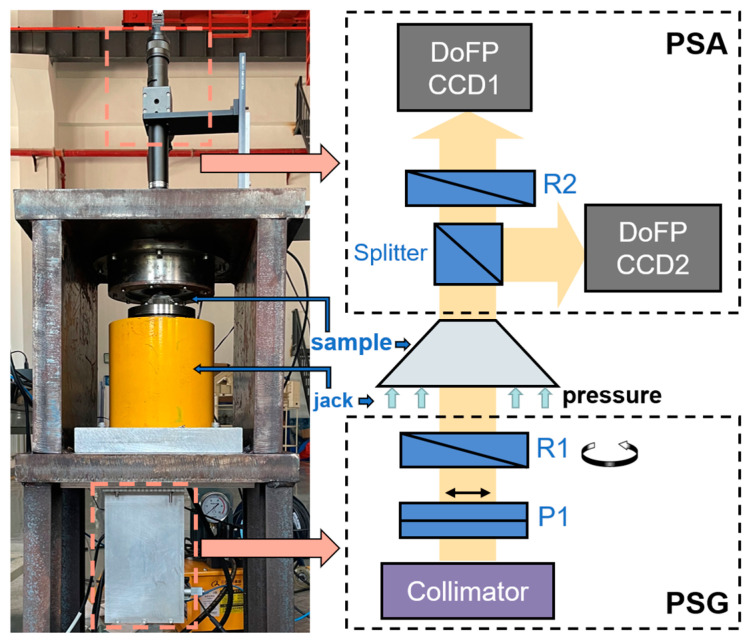
Experimental setup.

The experimental setup [20], as shown in Figure 2, can measure the Mueller matrix images of the samples subjected to different pressures. The light from the LED passes through the collimator to form a parallel monochromatic beam with a wavelength of 630 nm, a bandwidth of 10 nm and a diameter of 20 mm. This parallel beam passes sequentially through linear polarizer P1 (LPNIRB100, Thorlabs Inc., Newton, NJ, USA) and zero-order quarter-wave plate R1 (WPQ10E633, Thorlabs Inc., Newton, NJ, USA) mounted on a motorized rotation stage (PRM1/MZ8, Thorlabs Inc., Newton, NJ, USA) to generate a uniform incident light beam with controllable polarization states, which serves as polarization state generator (PSG). 

The light beam, then incident perpendicularly on the sample, goes through the sample, carries the information of the sample, and shoots out. The polarization state analyzer (PSA) is used to acquire the Stokes vector image of the output light beam. This PSA consists of a non-polarized beam splitter (CCM1-BS013/M, Thor-labs Inc., Newton, NJ, USA), a zero-order quarter-wave plate R2, and two 12-bit DoFP cameras (PHX050S-PC, Lucid Vision Labs Inc., Vancouver, BC, Canada) with resolution of 2448 × 2048 pixels, pixel size of 3.45 µm × 3.45 µm, photo response non-uniformity of 1.66%, linearity error max/min of 0.22%–0.50%. Although the specifications including the linearity of intensity of the cameras rely on the manufacturer’s testing report, there is no problem during the experiments in this work, and the final measurement error after the calibration is quite low as shown in the following text. The intensity of the polarization channel of each pixel is $I={\left[I_{0},I_{45},I_{90},I_{135}\right]}^{T}$, and the *S_out_* can be calculated by

(2) $$S_{\mathrm{out}}=pinv\left(A_{\mathrm{DoFP}}\right)\times I,$$

where *pinv*(*x*) is the pseudo-inverse of the matrix *x,* and the *A*_DoFP_ is the camera’s instrument matrix at this pixel obtained by calibration [22]. In PSA, the light beam is split into two beams by the beam splitter at a ratio of 50:50; the transmitted beam passes through R2 and is collected by DoFP camera CCD1, and the reflected beam is directly collected by DoFP camera CCD2, which is designed to measure the Mueller matrix. 

PSG generates the four different polarization states by rotating the orientation of R1 to be −45°, −19.6°, 19.6° and 45° [23], which are deliberately selected to minimize the random errors by reaching the smaller value of the number condition(CN) of the instrument matrix [24]. Accordingly, PSA acquires the Stokes vectors images of output light beam. For each image pixel, we combine the Stokes vectors of the incident light to form the 4 × *n* incident set [*S_in_*]_4×*n*_. Similarly, the output set [*S_out_*]_4×*n*_ can be obtained accordingly. Based on Equation (1), the Mueller matrix *M* of each pixel can be calculated from Equation (3) [25],

(3) $$M={\left[S_{\mathrm{out}}\right]}_{4\times n}\times pinv({\left[S_{\mathrm{in}}\right]}_{4\times n}),n>4.$$

Then, we can obtain the Mueller matrix image of the sample.

As shown in Figure 1, the former face of the sample is pressurized with a jack (RRH-1003, Yuli Electromechanical Equipment Group Co., Taizhou, China), which can maintain the given pressure on the sample for over 360 min. According to the pressures when the submersible working in the full sea depth and the regular working time [26], we conducted four experiment groups using four different samples, with pressures of 60 MPa, 90 MPa, 120 MPa, and 150 MPa, respectively. Notably, irreversible deformation occurs when the sample is subjected to a relatively large load, so a new sample is used for each pressure experiment. The Mueller matrix images are consecutively acquired for 360 min during each experiment group.

### 2.3. Calibration Method

To improve the measurement accuracy of the Mueller matrix, calibration has been carried out. We used a polarimeter (PAX1000VIS, Thorlabs, Newton, NJ, USA) as the standard device. The PSG generates a series of incident polarization states, each of which is measured by the polarimeter to ensure the accuracy of *S_in_*. After that, the light beam with known *S_in_* directly illuminates the PSA. Note that the four polarization states in [*S_in_*]_4×*n*_ are not included. Then, the so-called instrument matrix of PSA is obtained to transfer pixel values of the dual-DoFP camera to the Stokes vector of the illumination light. Finally, we evaluate the setup’s measurement error of the Mueller matrix. We use the air as the standard sample whose Mueller matrix is the unit matrix. For the measured 4 × 4 Mueller matrix images, the mean value of each element image is first obtained, and then a 4 × 4 matrix consisting of these mean values is obtained. The measurement error is the maximum of the absolute error between the elements of this 4 × 4 matrix and unit matrix, which is less than 0.01 for the current setup. The deformation and deviation of the sample after pressurization can distort the corresponding image pixels of the incident and outgoing light, leading to inaccuracies in the Mueller matrix measurement. In order to address this image distortion, we used projection transformation to recover the deformed image to its original form [20]. 

### 2.4. Parameters

The Mueller matrix contains abundant information of the sample’s physical properties [27], such as the microstructure, birefringence, etc.; the polarization parameters derived from the Mueller matrix help to explicitly express the meaningful correlation to the sample’s physical properties [28]. In this paper, Mueller matrix transformation (MMT) technique [29] is used to analyze the Mueller matrix images of the sample, and the rotational invariant *t*_1_ is obtained by Equation (4) to characterize the total linear anisotropy of the sample [30]. Additionally, the Mueller matrix polar decomposition (MMPD) method is used to decompose the Muller matrix into the depolarizer *M*_Δ_, the retarder *M*_R_ and the diattenuator *M*_D_; then, the parameter *R* characterizing the total retardance are calculated from Equations (5)–(6) [31].

(4) $$t_{1}=\frac{1}{2}\sqrt{{\left(m_{22}-m_{33}\right)}^{2}+{\left(m_{22}+m_{33}\right)}^{2}}$$

(5) $$M=M_{\Delta }M_{R}M_{D}=\left[\begin{array}{cc} 1 & {\vec{0}}^{T}\\ \vec{P_{\Delta }} & m_{\Delta } \end{array}\right]\left[\begin{array}{cc} 1 & {\vec{0}}^{T}\\ \vec{0} & m_{R} \end{array}\right]\left[\begin{array}{cc} 1 & {\vec{D}}^{T}\\ \vec{D} & m_{D} \end{array}\right]$$

(6) $$R=\arccos\left[\frac{1}{2}tr\left(M_{R}\right)-1\right]$$

To characterize the creep deformation of the sample, we employed the Pearson linear correlation coefficient to measure the degree of correlation between different images at constant pressure. A stronger correlation is indicated by a coefficient closer to 1, while a weaker correlation is revealed by a coefficient closer to 0. Equation (7) is used to calculate the two-dimensional Pearson linear correlation coefficient *r*, where *A* and *B* are the pixel matrices corresponding to the two compared images, and $\bar{A}$ and $\bar{B}$ respectively represent their pixel averages [32].

(7) $$r=\frac{\sum_{m}\sum_{n}\left(A\left(m,n\right)-\bar{A}\right)\left(B\left(m,n\right)-\bar{B}\right)}{\sqrt{{\left(\sum_{m}\sum_{n}\left(A\left(m,n\right)-\bar{A}\right)\right)}^{2}{\left(\sum_{m}\sum_{n}\left(B\left(m,n\right)-\bar{B}\right)\right)}^{2}}}$$

## 3. Results

### 3.1. Polarization Parameter Images

**Figure 3:**
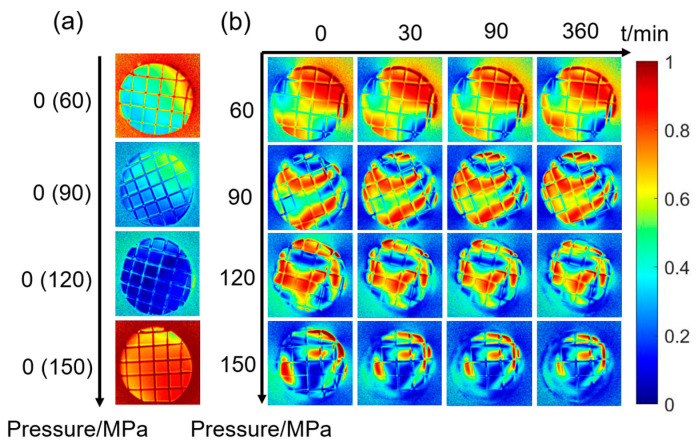
Parameters *t*_1_ (**a**) unpressurized and (**b**) at different pressures and times.

The *t*_1_ images before pressurization are displayed in Figure 3a, where 0 indicates that it is the image of sample that has not been pressurized, and the value of the pre-pressurization is shown in the brackets. One can see from Figure 3a, the circular area at the center of each image is the image of the latter surface of the sample, which appears distinct from the background and other parts of the image. For convenience, in this paper, when the polarization parameter image is mentioned, only the image of the latter surface of the sample is considered. 

As shown in Figure 3a, the distribution of the *t*_1_ images appears relatively uniform. Figure 3b presents the *t*_1_ parameter during the creep for 360 min under the pressures of 60 MPa, 90 MPa, 120 MPa, and 150 MPa. It has been known that after pressurization, the internal stress and strain changed with the magnitude of the load and the time [33]. From Figure 3b, the inhomogeneity of the *t*_1_ images becomes more obvious with higher pressure, indicating an increase in both stress and strain of the sample [34,35]. Over time, a similar tendency can also be observed; and in detail, it seems that the edges dynamically shift as time increases, which makes the distribution becomes richer of textures. For example, for the top portion of 90 MPa images, the red area, whose value falls in the range of 0.8–1, gradually enters the region of view with the time; the shape of this area appears as a small dot at *t* = 0 min, whereas it eventually transforms into a large red arc at *t* = 360 min. Moreover, for the images at 120 MPa, we can see that an entire red area in the middle part of the image slowly shrinks towards the center while the image becomes more distorted. The increase of the inhomogeneity of *t*_1_ images may indicate that the strain of the sample is increasing since the stress here stays the same all the time.

**Figure 4:**
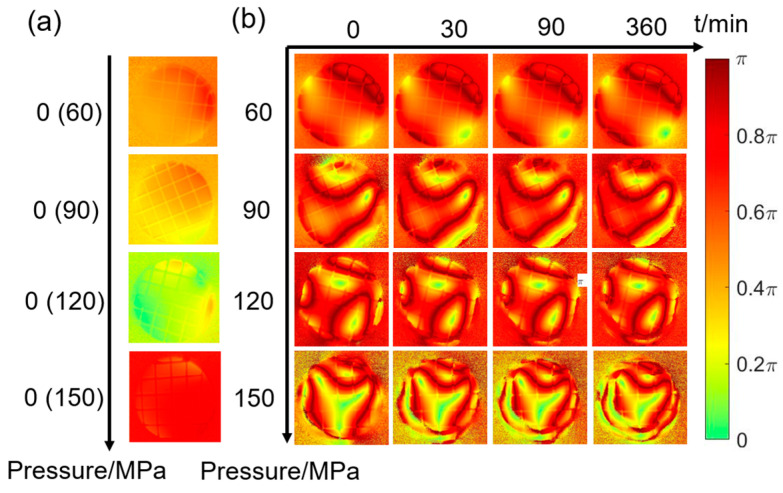
*R* (**a**) unpressurized and (**b**) at different pressures and times.

The *R* images of the sample before pressurization are shown in Figure 4a, all of which look uniform. Figure 4b shows the *R* images during creep for 360 min at 60 MPa, 90 MPa, 120 MPa, and 150 MPa. It can be observed that there are crimson annular regions in each graph, which are split by the curves whose curvature and density both go up with increasing pressure and time. This indicates that *R* can also characterize the creep effectively. It should be noted that the retardance *R* is subjected to a π period [36], which affects the experimental results and needs further study.

### 3.2. Unwrapped R Images

Looking at *R* images in Figure 4, it can be seen that the image exhibits noticeable inhomogeneity, and a series of crimson arcs with different shapes can be seen inside. From the physical origin, *R*, calculated by the MMPD method, characterizes the equivalent retardance of the sample, which is always wrapped between 0 and π. Since structural changes in the sample are usually spatially continuous rather than abrupt, the presence of crimson lines in *R* images could be attributed to *R*’s wrapping. 

**Figure 5:**
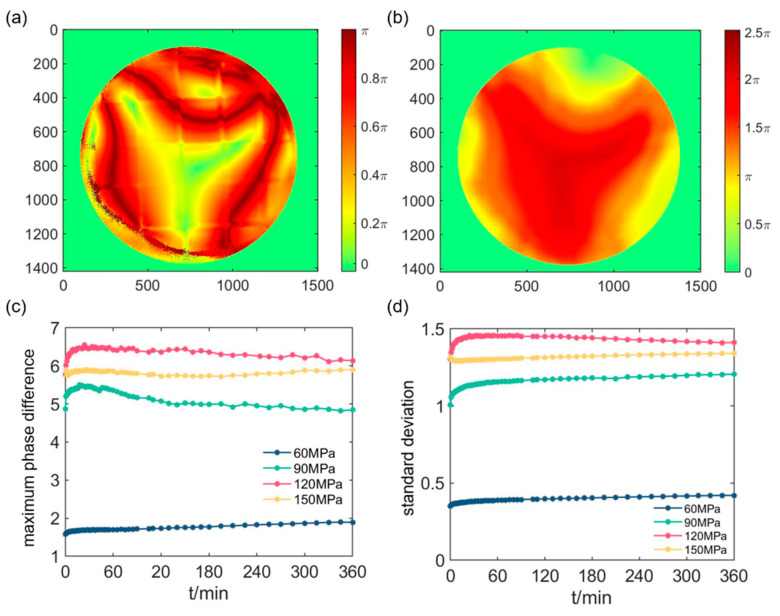
(**a**) *R* image and (**b**) *R*′ image at 150 MPa. (**c**) Maximum phase difference for *R*′ images versus creep time and (**d**) standard deviation for *R*′ images versus creep time at pressures of 60 MPa, 90 MPa, 120 MPa and 150 MPa.

In order to restore the retardance to its true value, a two-dimensional retardance unwrapping method is used to unwrap the *R* images [37]. The gradient is calculated from the center point of the image to the periphery, and if a position is judged to be cross-period, it will be added or subtracted by a multiple of π accordingly. This process ensured that the retardance values obtained from adjacent pixels are continuous. The parameter *R*′ is defined as the unwrapped *R*. The first moment (*t* = 0 min) *R* image at 150 MPa after the pressurization is shown in Figure 5a, and its corresponding *R*′ image is shown as Figure 5b. 

The difference between the maximal and minimal values for each of the *R*′ images, that is *R*′_diff_, versus the creep time is shown in Figure 5c. *R*′_diff_ represents the largest retardance carried by the penetrating light, which originates from the birefringence of the sample; accordingly, we can infer the distribution of the additional birefringent index from the creep if the geometrical length of the light path can be known firstly [9]. Similarly, the standard deviation for each *R*′ images, that is *R*′_std_, versus the creep time for is shown in Figure 5d, which characterizes the heterogeneous of *R*′ images.

At 60 MPa, *R*′_diff_ rises faster at the first times and then grows slowly. However, at 90 MPa, *R*′_diff_ show an upward trend to its maximum for the first times and then decline slowly, which is similar for those at 120 MPa. At 150 MPa, the similar rising of *R*′_diff_ appears firstly but then it experiences a complex changing as the creep time increases. In addition, except for those at 150 MPa, *R*′_diff_ totally has the larger values at the larger pressures, which is consistent with the fact that the higher pressures generate greater birefringence. As shown in Figure 5d, *R*′_std_ of *R*′ images also shows a rising at the first times but with the successive slow growing or declining for the different pressures. Additionally, *R*′_std_ totally has the larger values at the higher pressures, except of those at 150 MPa. 

### 3.3. Correlation Coefficient of t_1_ Images 

To further investigate the ability of polarization parameters to characterize samples creep, we compare the *t*_1_ and *R* images in Figure 3 and Figure 4 with the pressure and time, respectively. For the images of the latter surface, in Figure 3 and Figure 4, their sizes become small continuously as the creep time increases, especially those at 150 MPa pressure. In each image, there is a clear area in its middle where the auxiliary lines are visible, while the part outside this clear area looks blurred as the auxiliary lines disappear. Furthermore, as the creep time increases, the clear area shrinks inward progressively, while the blurred area expands. Therefore, it is recommended to consider the clear area firstly in the following quantitative analysis. 

**Figure 6:**
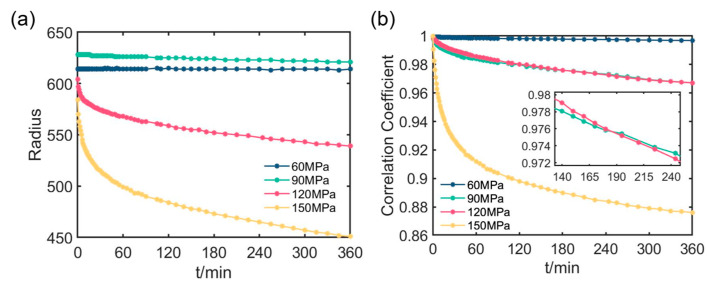
(**a**) Radius versus creep time and (**b**) correlation coefficient versus creep time at 60 MPa, 90 MPa, 120 MPa and 150 MPa.

For each polarization parameter image, we use the radius of the minimal circle covering the clear area to represent the size of the clear area. Figure 6a collects the size of the clear area with different pressures and creep times. Obviously, the size of the clear area decreases with increasing pressure. The sizes of the clear area at 60 MPa change least as the creep time increases, and those at 90 MPa change slightly, but those at 120 MPa change significantly and those at 150 MPa change most. Finally, the clear area reaches its minimum size at the last creep time. Note that the sizes of the clear area after the pressurization are not consistent with the pressures, due to differences among individual samples. 

To calculate the correlation, we selected the size of the clear area at the last creep time and chose the same area for each *t*_1_ image. The first moment image, captured immediately after pressurization (*t* = 0 min), was used as the reference image, and its correlation coefficient with the images at all subsequent times are evaluated. Figure 6b collects all the correlation results, which show that the correlation coefficient decreases with creep time; this decline is more rapid in the early stage but tends to decrease linearly in the later stage. 

Additionally, it is noteworthy that the correlation coefficient declines faster at higher pressures than at lower ones. Specifically, after 360 min at 60 MPa, the correlation coefficient decreased from 1 to 0.9968, only reducing 0.0032, indicating that the creep deformation of the sample under small pressure is not obvious. However, after 360 min at 150 MPa, the correlation coefficient decreased from 1 to 0.8761, a change of 0.1239, over 38 times larger than at 60 MPa. which can be inferred that the creep deformation of the sample will occur significantly under high pressure. Obviously, large pressure can cause significant creep of the sample. 

In addition, one notices that during the creep, the correlation coefficients at 120 MPa are similar with those at 90 MPa. However, as shown in the inset of Figure 6b, the correlation coefficients at 120 MPa decline faster than those at 90 MPa, which is consistent with the expectation that higher pressure would lead to more serious creep [33]. However, the differences between the samples discussed later should be taken into account when considering the practical usage of these polarization parameters.

In conclusion, the findings discussed above are consistent with the creep law of PMMA materials [38], representing that the correlation coefficient of polarization images has the potential to serve as an indicator of the overall creep of an observation window under constant pressure.

When the sample is subjected to an upward load, it is squeezed and deformed with the window seat, and subsequently, a deviation occurs between them. Under the constant load, sliding, diffusion and dislocation happen with the movement of particles in the sample [39], resulting in further changes in the internal microstructure, manifesting themselves macroscopically as creep. The internal stress-strain situation changes with time, and the degree of deformation and deviation gradually increases.

Using Mueller matrix imaging, we can not only visualize the spatial distribution and temporal changes about the observation window’s creep using *t*_1_ and *R* images but also quantitatively evaluate the creep through parameters such as *R*′ and *r*. This shows the capability of the proposed method for the observation window’s creep monitoring.

## 4. Finite Element Analysis (FEA) of Window’s Creep 

### 4.1. FEA Model

**Figure 7:**
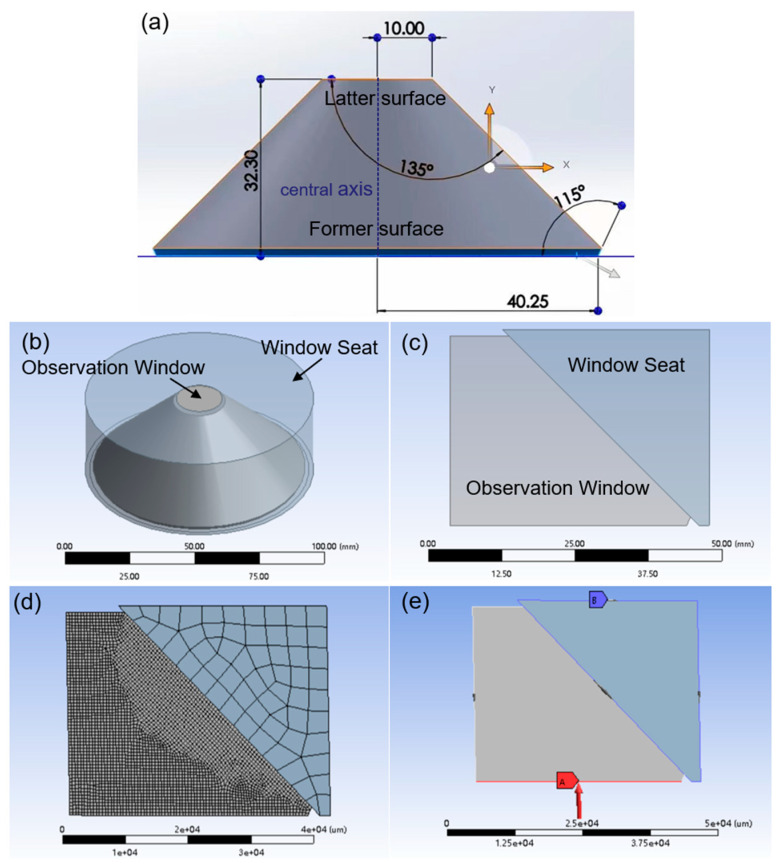
(**a**) Geometry of observation window (unit: mm), (**b**) 3D layout of the observation window and window seat, (**c**) 2D layout of observation window and window seat (unit: mm), (**d**) mesh generation and (**e**) static structure of the model. (unit: μm).

To demonstrate the validity of the polarization parameters, we used ANSYS (version 2021) to establish a finite element model of the PMMA observation window and the titanium window seat, with the geometry shown in Figure 7a and the physical parameters listed in Table 2. The three-dimensional (3D) layout of the observation window and its seat is shown in Figure 7b. Considering the rotational symmetry of the structure, a two-dimensional (2D) model after sectioning is taken for the simulation, which is shown as Figure 7c. Then, we set the observation window’s mesh size to 0.5 mm and the window seat’s mesh size to 3.3 mm, with the mesh generation shown in Figure 7d. Figure 7e displays the static structure of the model, with fixed support surrounding the window seat and a uniform vertical pressure applied to the former surface of the observation window. The friction between the observation window and the window seat is set as frictional contact with the friction coefficient of 0.05. A modified time hardening model embedded in ANASYS is used to analyze the creep of the observation window, and the internal strain of the material is computed as follows:

(8) $$\varepsilon =\frac{C_{1}\sigma^{C_{2}}t^{C_{3}+1}e^{-C_{4}/T}}{\left(C_{3}+1\right)},$$

where *σ* is the current stress, *T* is the time, and *C*_1_, *C*_2_, *C*_3_, *C*_4_ are constants related to the creep properties of the material. Referring to the engineering data of creep parameters of the submersible’s observation window [13], *C*_1_ = 3.5 × 10^−5^, *C*_2_ = 0.98, *C*_2_ = −0.89, *C*_4_ = 0, and the ambient temperature of the simulation is set to 25 °C.

### 4.2. FEA Results

**Figure 8:**
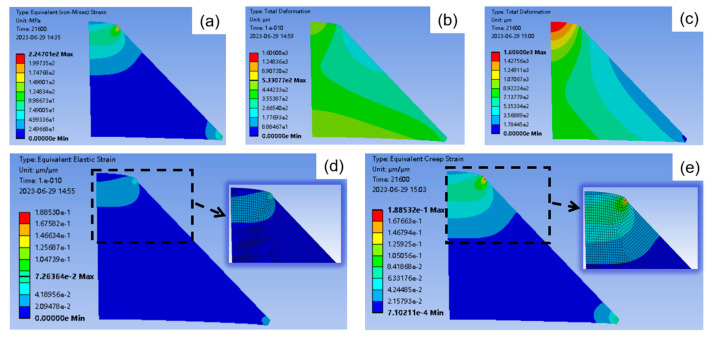
Finite element analysis results of observation window under 120 MPa pressure: (**a**) Von-Mises stress nephogram with *t* = 360 min, (**b**) strain nephogram with *t* = 0 s, (**c**) strain nephogram with *t* = 360 min, (**d**) equivalent creep strain nephogram with *t* = 1 min, (**e**) equivalent creep strain nephogram with *t* = 360 min.

In the creep simulation, the pressure is set to 120 MPa for 360 min. The von Mises stress distribution of the observation window is shown in Figure 8a. From the former surface to the latter surface, the stress’s distribution looks layer-like structure, and for the layers close to the surfaces, the stress is small; however, for the internal layers close to the latter surface, the stress is high to the maximum. Moreover, the maximal point of the stress occurs at the junction between the latter surface of the observation window and the window seat due to the structural discontinuity and the substantial difference in stiffness between the materials [12]. 

The total deformation at the initial stage (*t* = 0 min) of creep and after 360 min creep are, respectively, shown as Figure 8b,c. After the creep, the total deformation of the observation window becomes noticeable. The latter surface extrudes upwards, and its total deformation close to the central axis reaches the maximum. This corresponds to the shrinkage of the clear area observed in *t*_1_ and *R* images shown in Section 3.1. 

The equivalent creep strain distribution nephograms of the observation window for *t* = 1 min and for *t* = 360 min are shown in Figure 8d,e, respectively. The areas near the latter surface are selected to enlarge as the inserted images. From these two inserted images, one can see that the strain concentration of the latter surface spreads from the maximal point of the stress in Figure 8a, to the center area over time, which is consistent with the polarization parameter images in Section 3.1. For instance, the red edges of the *t*_1_ images gradually spread towards the center in Figure 3, and the curvature of the crimson ring in the *R* images increases over time in Figure 4. In addition, from Figure 8d,e, the creep effect makes the strain delamination of the observation window more serious. 

**Figure 9:**
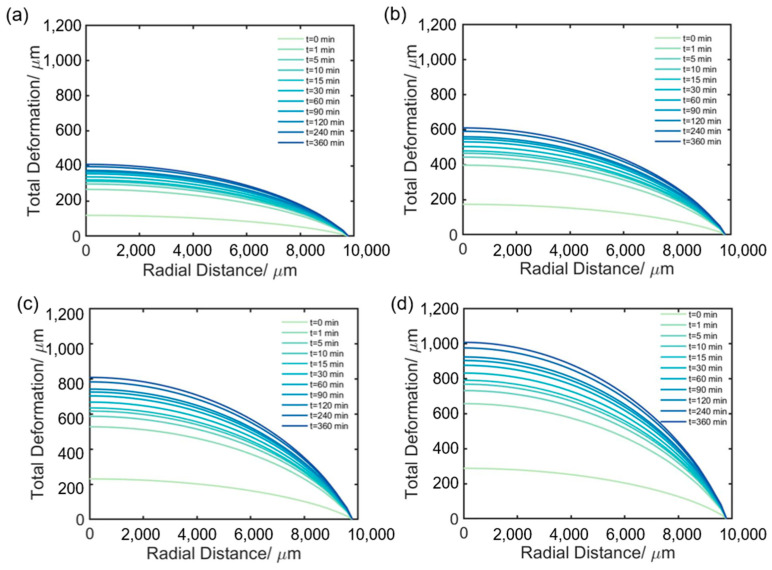
Total deformation distribution versus creep time for the latter face with different pressures: (**a**) 60 MPa, (**b**) 90 MPa, (**c**) 120 MPa, (**d**) 150 MPa.

Creep simulations are carried out by subjecting the observation window to different pressures of 60 MPa, 90 MPa, 120 MPa, and 150 MPa for 360 min. Figure 9a–d shows the total deformation of the latter surface changes as the creep time increases under the different pressures. As seen from these figures, the upward extrusion of the latter surface increases with time and pressure. Notably, the center of the latter surface looks relatively flat, and the edge looks steep. This steep edge greatly deflects the propagation path of light, thereby increasing the numerical aperture (NA) of the observation windows. If the NA is so large that the light cannot be received by PSA whose NA is fixed, the edge areas of the latter surface would not be effectively imaged by the cameras, resulting in blurry images as seen in *t*_1_ and *R* images of Section 3.1. Moreover, in Figure 3 and Figure 4, larger blurred areas are observed for higher pressure or the longer creep times, which is consistent with the steeper edges shown in Figure 9a–d.

**Figure 10:**
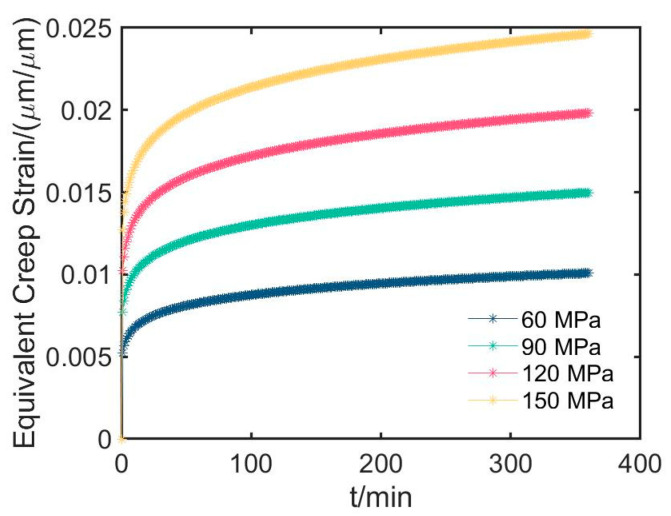
Equivalent creep strain for different pressures versus creep time.

The average equivalent creep strains for all creep time are evaluated and shown in Figure 10. During the early stage of creep, the strain increases at a rapid rate, and then the constant rate stage is reached after a certain inflection time. This presents that the first stage and the second stage of creep mentioned in Section 2.1 are demonstrated, and corresponds to the first steep, and then slowly trends in the curves in Figure 5 and Figure 6. The larger pressure results in the larger average equivalent creep strain and the shorter inflection time, which is consistent with the creep characteristics of the PMMA materials [40]. There are some similarities between Figure 10 and Figure 6, such as the large pressure introducing the short inflection time. However, there are also some obvious discrepancies between the two figures. These differences may be attributed to the physical variations in used parameters as well as the impact of sample differences and complex conditions that could not be covered in the simulations.

Recall that in Figure 6b, the correlation coefficients at 90 MPa are close to those at 120 MPa, although the latter declines faster than the former. From Figure 9 and Figure 10, one can see that both the deformation and equivalent creep strain at 90 MPa look less serious than those at 120 MPa. The mismatching between the experiments and the simulations may be due to the fact of the difference between the samples. It should be noted that the experiments were conducted independently using different samples. Moreover, as seen in Figure 6a, the selected radii at 90 MPa are larger than those both at 120 MPa and at 60 MPa, which indicates that the difference between the samples are not neglected. Still, the declining of the correlation coefficients in Figure 6b for all pressures can be explained by the temporal successive deformation and average equivalent creep strain shown in Figure 9 and Figure 10.

Overall, the finite element analysis results show the deformation of the observation window under the pressure and the stress-strain transfer processes inside it during creep. These results offer valuable insights into the characterization capability of the Mueller matrix images for the creep in the scaled-down samples.

## 5. Discussion

### 5.1. More Potential Polarization Parameters


(9) $$\psi =\frac{1}{2}\arctan\left[\frac{M_{R21}-M_{R12}}{M_{R11}+M_{R22}}\right]$$

(10) $$b=\frac{1}{2}\left(m_{22}+m_{33}\right)$$

(11) $$A=\frac{2bt_{1}}{b^{2}+t_{1}^{2}}$$

(12) $$D=\sqrt{{\left(m_{24}+m_{42}\right)}^{2}+{\left(m_{43}+m_{34}\right)}^{2}}$$

**Figure 11:**
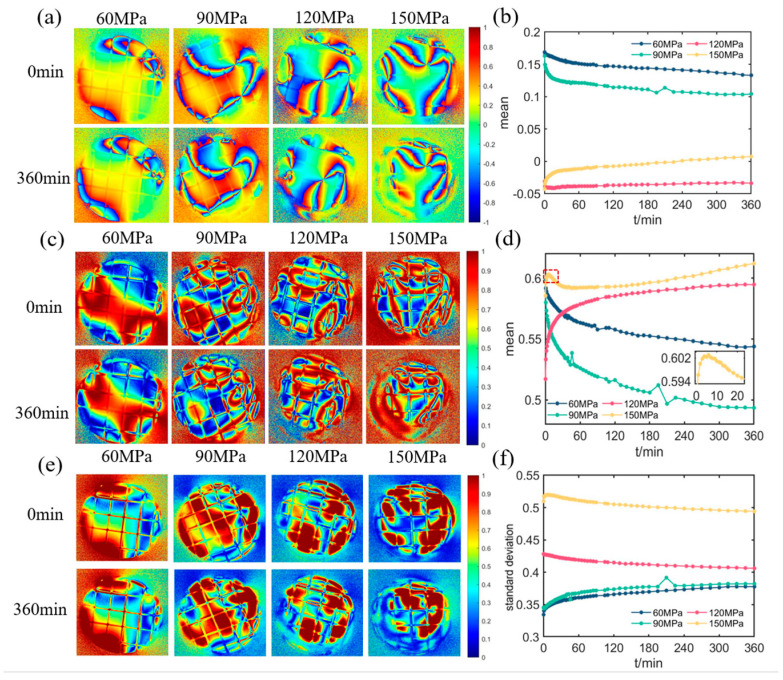
(**a**) *ψ* images and (**b**) their mean values versus creep time. (**c**) *A* images and (**d**) their mean values versus creep time. (**e**) *D* images and (**f**) their standard deviation versus creep time.

Besides *t*_1_ and *R*, we also find other polarization parameters that have the ability, more or less, to characterize the creep of the sample. One such parameter is the optical rotation angle *ψ*, which is calculated using Equation (9). Figure 11a,b shows the corresponding images and the mean values with the creep times, respectively. The *ψ* images more prominently show the crimson ring than *R* images. The mean value tends to change in the opposite direction for the high pressure of 150 MPa compared to the other three lower pressures, suggesting the existence of some critical value for the sample property transition below 150 MPa. 

The anisotropy parameter *A* after normalization of the *t*_1_ combined with the *b*, is calculated by Equations (4), (10) and (11) [41]. Figure 11c shows the *A* images at different pressures for *t* = 0 min and *t* = 360 min. The *A* mean values as the creep time are shown in Figure 11d. As the creep time increases, the mean values at 120 MPa and 150 MPa change contrarily to those at 60 MPa and 90 MPa, which may imply that *A* is more sensitive to the sample’s properties than *ψ*. There is also a noticeable spike in the early stage of the creep at 150 MPa, as shown in the inserted image in Figure 11d, which may be linked to the high pressure causing abrupt changes in the sample’s performance [42]. 

*D* is the transposed asymmetric parameter of the Mueller matrix, and it is calculated using Equation (12). The corresponding images and their standard deviations versus the creep time are shown in Figure 11e,f, respectively. The standard deviation is correlated with the pressure, which lets *D* win out the other parameter mentioned above. According to Ref. [43], nonzero values of D indicate the presence of a layer-like structure in the sample. Therefore, this can be seen in Figure 11e, where the nonzero pixel values signify the existence of multiple layers in the sample, which can be supported by the complex layered structure from the simulation results presented in Figure 8.

From Figure 11, one can see that these parameters have some advantage to characterize the creep of the samples with the different pressures, and their physical meanings also can be explained by the simulations in Section 4.2 or the literature. However, they still need to be deeply mined or carefully combined to monitor the observation windows’ creep. 

From the experimental results, it is observed that the changes of these characteristic parameters for the different pressures are much larger than those during the creep, which means that the creep monitoring needs more sensitive parameters to character its temporal changes. Additionally, since the creep will last for a relatively long time, it requires the parameters more sensitive to the creep but insensitive to the ambient factors than those in the pressurization. Therefore, the creep monitoring in this work is intrinsically different and more challenging than the previous research in Ref. [20], although it benefits from the latter. 

### 5.2. Future Work

Many effects contribute to the difference patterns between the samples before the pressurization, such as the non-uniformness of the materials, manufacturing errors and the uncertainty during the sample replacement. Nevertheless, we can still demonstrate the feasibility of the proposed method. For each sample, we continuously record the change of patterns under a given pressure to reflect the creep process. For samples with different pressures, although the details of the patterns are quite diverse, statistical methods such as radius size, correlation coefficient, mean, and standard deviation are used to quantitatively compare the influence of pressure on the creep.

We notice that in Figure 6a, the radius of the clear area of *t*_1_ images decreases as the creep time increases. Combining with the simulated deformation of the surface or extrusion of the windows in Figure 9, it is possible that the numerical aperture of the window gradually increases with the creep time and exceeds the limit of the receiving optical system in PSA, which leads to the decreasing of the radius of the clear area. Meanwhile, as shown in Figure 6b, when we fix the area size to be the minimal clear area of *t*_1_ images, the correlation coefficient of the temporal image with the reference (original) image decreases with the creep time, which means the image pattern gradually deviates from the original image. From Figure 6, one can see that the image features of the polarization parameters, including the geometry or the texture, would also be the effective clues to characterize the window creep. Future work would include this part when applying the proposed method to the in-situ monitoring of the window creep.

During the creep, the clear areas in polarization parameter images gradually shrink while the blurred areas gradually expand. Only the central part of the clear areas is selected to analyze in this work, but the other parts are ignored. However, from the simulations, we know that the maximal point of the stress is located at the edge area of the sample which coincides with the blurred areas in the images. By increasing the NA of PSA, it may be possible to expand the clear areas. Furthermore, more advanced and sophisticated optical systems, such as through the application of zoom lens [44] and adaptive optics [45], are required to be adopted to improve the imaging quality and make the blurred areas image clearly. Moreover, the polarization imaging can be combined with the other imaging methods such as the spatial frequency domain imaging [46,47] to obtain the internal stress distributions of windows during the creep.

Experimental results in Figure 3, Figure 4, Figure 5 and Figure 6 and 11, show that the temporal changes of the polarization parameters have a strong correlation with the classical creep description. For example, as the time increases, the proposed characteristic parameters change rapidly at the early stage and then change slowly at the latter stage, which is quite similar with the primary creep and steady-state creep described in Ref. [21]. FEA is well known and believed to be a powerful tool to study the geometric deformation and stress distribution during the creep. The FEA simulations build the logic relationship between the creep and the characteristic parameters by explaining the optical effects in physical origin. For example, the deformation in Figure 9 deflects the propagation direction of the light, and the internal stress distribution results in the birefringence inside the window. All these optical effects can be characterized by the characteristic parameters including the radius of the clear area, and polarization parameters, such as *R*′, *t*_1_, *ψ*, *A*, *D*, etc. These indicate that the characteristic parameters physically describe the changes of the windows during the creep, which ensures the effective monitoring.

The method proposed in this paper still does not explicitly build a direct or empirical relationship between polarization parameters and physical parameters like stress and strain. However, since the Mueller matrix encapsulates the total polarization properties of the sample, the sophisticated design of the polarization parameters would be included in the future work. For example, deep learning and machine learning are applied to this optical process [48]. Polarization parameters can be fused and simplified into one or more visual parameters (such as refractive index, stress and strain) so as to determine whether there is a safety problem in the window at this moment and predict the state of the next period. 

This study aims to monitor the creep deformation of the submersible window, and a series of images and parameters with characterization capabilities are obtained. However, the current experimental method involves using a jack to pressurize a scaled-down sample in the air, which is still significantly different from the real underwater environment. In the future, it might be feasible to design an in-situ device that can monitor the creep of the submersible observation window working in the deep sea. 

## 6. Conclusions

The observation window’s creep under pressure can adversely affect the load-bearing capacity and safety performance of the submersible. In this paper, a conceptual setup is built to monitor the creep of the observation window by continuously acquiring the sample’s Mueller matrix images. The characteristic polarization parameters calculated from the Mueller matrix images, such as *t*_1_ and *R*, are proven to characterize the deformation of the observation window and monitor the observation window’s creep. We also derived some additional polarization parameters, such as the correlation coefficient *r* describing the tendency of the creep and the unwrapped retardation *R*′ reflecting information about the birefringence index during the creep. Then, the finite element analysis is employed to help explain the experimental results and validate the monitoring’s reliability. More polarization parameters are discussed to monitor the sample’s creep. In summary, this work proposes an effective method for monitoring the creep of the observation window which can potentially become a valuable tool for enhancing the safety and success of submersibles in deep-sea applications.

## Figures and Tables

**Table 1:** Physical properties of the sample [20].

Properties	Value
Density/ ($g/{\mathrm{cm}}^{3}$)	1.186
Tensile Modules/ GPa	3.13
Yield Strength/ MPa	129
Poisson’s ratio	0.37
Refractive index	1.49

**Table 2:** The physical properties of the finite element analysis.

Properties	Value
Observation Window’s Density/ (g/cm^3^)	1.186
Observation Window’s Tensile Modulus/ GPa	3.13
Observation Window’s Poisson’s ratio	0.37
Window Seat’s Density/ (g/cm^3^)	6.85
Window Seat’s Tensile Modulus/ GPa	200,000
Window Seat’s Poisson’s ratio	0.3
Friction Coefficient	0.05

## Data Availability

Not applicable.
